# Current understanding of the role of Adipose-derived Extracellular Vesicles in Metabolic Homeostasis and Diseases: Communication from the distance between cells/tissues

**DOI:** 10.7150/thno.42167

**Published:** 2020-06-12

**Authors:** Chun-Jun Li, Qian-Hua Fang, Ming-Lin Liu, Jing-Na Lin

**Affiliations:** 1 Department of Endocrinology, Health Management Center, Tianjin Union Medical Center, Nankai University Affiliated Hospital, Tianjin, 300121, P.R. China.; 2 Department of Dermatology, Perelman School of Medicine, University of Pennsylvania.; 3 Corporal Michael J. Crescenz VA Medical Center, Philadelphia, PA 19104, USA.; 4 Shanghai National Research Centre for Endocrine and Metabolic Diseases, State Key Laboratory of Medical Genomics, Shanghai Institute for Endocrine and Metabolic Diseases, Ruijin Hospital, Shanghai Jiao Tong University School of Medicine, Shanghai, China.

**Keywords:** Extracellular vesicles, inflammation, adipose tissue, obesity, metabolic disease

## Abstract

Extracellular vesicles (EVs) including exosomes, microvesicles (MVs), and apoptotic bodies, are small membrane vesicular structures that are released during cell activation, senescence, or programmed cell death, including apoptosis, necroptosis, and pyroptosis. EVs serve as novel mediators for long-distance cell-to-cell communications and can transfer various bioactive molecules, such as encapsulated cytokines and genetic information from their parental cells to distant target cells. In the context of obesity, adipocyte-derived EVs are implicated in metabolic homeostasis serving as novel adipokines. In particular, EVs released from brown adipose tissue or adipose-derived stem cells may help control the remolding of white adipose tissue towards browning and maintaining metabolic homeostasis. Interestingly, EVs may even serve as mediators for the transmission of metabolic dysfunction across generations. Also, EVs have been recognized as novel modulators in various metabolic disorders, including insulin resistance, diabetes mellitus, and non-alcoholic fatty liver disease. In this review, we summarize the latest progress from basic and translational studies regarding the novel effects of EVs on metabolic diseases. We also discuss EV-mediated cross-talk between adipose tissue and other organs/tissues that are relevant to obesity and metabolic diseases, as well as the relevant mechanisms, providing insight into the development of new therapeutic strategies in obesity and metabolic diseases.

## Introduction

The prevalence of obesity has increased dramatically around the world 1. It is estimated that more than 1.9 billion adults are overweight, of which over 650 million are obese 2. Obesity is a major problem in children with 41 million children under the age of 5 and over 340 million children and adolescents aged 5-19 reported to be overweight or obese in 2016 2. If the current trend continues, the number of overweight or obese young children is expected to reach 70 million by 2025 3. Obesity has become a serious public health concern in the 21st century due to the rapid increase in its prevalence and the negative impact of its complications on human health 4. Excess fat accumulation usually leads to various metabolic disorders, including insulin resistance (IR), type 2 diabetes mellitus (T2DM), and non-alcoholic fatty liver disease (NAFLD), resulting in a significant decrease in life expectancy as well as the quality of life 5, 6.

Adipose tissue (AT) was initially regarded as a type of tissue storing excess nutrients. However, recent studies demonstrated that AT could function as an endocrine organ, which secretes various adipokines, such as leptin, adiponectin, visfatin, resistin, and adipsin 7-9. These AT-derived adipokines can serve as mediators to regulate the function of other metabolic organs 7-9. Besides the above soluble mediators, extracellular vesicles (EVs), the subcellular membrane structures, have been shown to regulate pathophysiological conditions of other metabolic organs as insoluble mediators 10-12. EVs derived from the adipose tissue (AT-derived EVs) are distinct from traditional soluble adipokines and can modulate specific target cells because of their bio-active cargos 13. The role of EVs in human metabolic physiology and pathology has attracted increasing attention in the past few years. Given the involvement of EVs in various metabolic diseases, emerging information in this filed may provide insights into the development of potential new therapeutic strategies in obesity-related human diseases. In this review article, we will summarize recent advances from basic and translational studies of EVs and focus on their role in obesity and metabolic diseases.

## Extracellular Vesicles

EVs, membranous subcellular structures with lipid bilayers and cytoplasmic components, are released from their parental cells in a highly regulated manner 11, 12. The EV-associated bioactive cargos include proteins, lipids, multi-molecular complexes, and nucleic acids (DNA, RNA, siRNA, microRNA, and lncRNA), many of which have been shown to modulate gene expression and signaling pathways in target cells. EVs can be released from almost all types of cells, including normal, malignant, and senescent cells, and particularly cells undergoing several types of programmed cell death, like apoptosis, pyroptosis, and necroptosis 14-15. EVs exist in various solid organs/tissues and biological fluids, such as urine, blood, breast milk, semen, and amniotic fluid 16, and are involved in physiological functions. In pathologic conditions, such as cancer, infectious, and metabolic diseases, elevated levels of EVs have been observed 17-19. Based on the differences in size and biogenesis, EVs can be broadly categorized into exosomes, microvesicles (MVs), and apoptotic bodies 20. Various EV isolation methods have been developed by exploiting the differences in size, buoyancy, or surface membrane marker expression 12.

Exosomes, the smallest EVs with the size range of 30 - 100 nm in diameter, are generated from the endosomal compartment and released into the extracellular space as nano-sized membrane vesicles. Pioneering studies from Harding et al. and Pan et al. suggested that exosomes arise by budding from the intracellular endosomal membranes 21-23. At least three molecular mechanisms have been identified that are involved in the assembly and loading of EVs, like the endosomal sorting complex required for transport (ESCRT) machinery, sphingolipid ceramide, and tetraspanin CD63 24, 25. These mechanisms may regulate different bio-active cargos packed in diverse exosomes from a variety of cell types under various pathophysiological conditions. Most exosomes contain abundant lipids, including cholesterol, sphingolipids, and probably phosphatidylserine (PS) 26. Further investigations of the molecular architecture and the underlying mechanisms in the assembly and loading of EVs are needed.

MVs (also called microparticles), ranging from 100 to 1,000 nm in diameter, are produced from the cell surface plasma membrane by a budding process. MVs, first described in 1967 by Peter Wolf, originated from platelets for their prothrombotic function 27. Exposure to phosphatidylserine (PS) from the inner surface of the cell membrane is a common feature of MVs from activated or apoptotic cells 10, 11, 28. MVs carry bioactive molecules from the membrane, cytoplasm, nucleus, and other organelles 10, 11. Our previous studies have reported that cell membrane MMP-14, ADAM10/17, or nuclear HMGB1 could be released with MVs from human macrophages or neutrophils when exposed to tobacco smoke extract probably through apoptosis induction 29, 30. In addition, EVs have recently been demonstrated to be derived from cells undergoing other types of programmed cell death, such as necroptosis and pyroptosis 13, 31.

In necroptic cells, EVs release is regulated by activation of receptor-interacting protein kinase-3 (RIPK3) and phosphorylation of mixed lineage kinase domain-like (MLKL) protein 32. Like apoptotic cells, necroptotic cells also externalize PS on the outer plasma membrane after the membrane translocation of phospho-MLKL 32, which is enclosed and released with EVs, as a mechanism for the self-restricting action of cells from the necroptotic activity of MLKL 33. In contrast, inhibition of MLKL phosphorylation could protect cells from necroptotic cell death, resulting in reduced EVs release and restricted inflammatory response 34. Pyroptosis is a highly inflammatory form of programmed cell death, which is characterized by the release of interleukin-1β (IL-1β) or interleukin-18 (IL-18) 35. Pyroptotic cells release these cytokines from cytoplasm through cell membrane pore molecule gasdermin D (GSDMD), the mature form of which is cleaved by caspase-1 and caspase 11/4/5 36-40. Pyroptotic cells release cytokine-containing MVs when encountered by various pathological stimulations, including stroke, heart attack, or cancer 41. Furthermore, pyroptosis could also serve as a novel driver of the inflammatory response in liver injury and fibrosis 42.

These studies have demonstrated that EVs derived from apoptotic, necroptotic, and pyroptotic cells may have different functions in target cells, probably because of the differences in MV-associated bioactive cargos that are released by different mechanisms. The bioactive molecules of EVs may be involved in both physiological and pathological processes, and contribute to various human diseases, including obesity and metabolic diseases.

Besides programmed cell death, recent studies showed that MVs could also be released by senescent cells 15, 43. Obese individuals have increased levels of circulating pro-inflammatory cytokines with age that might be secreted by senescent cells, leading to the development of metabolic diseases 44. Targeting human senescent fat cell progenitors could suppress the release of activin A, an adipokine that regulates energy balance and insulin insensitivity 45. Senescence-associated EV secretion from cancer cells was first described by Lehmann et al. 15. So far, DNA-damaging reagents, irradiation, serial passaging, and oncogenic Ras expression have been shown to contribute to the secretion of EVs from senescent cells 46. Additionally, harmful molecules produced during stress or pathological conditions could be eliminated from senescent cells by the release of EVs as “dumping garbage” to maintain cellular integrity and homeostasis 47. Interestingly, the levels of extracellular eNAMPT (nicotinamide phosphoribosyltransferase) declined with age in mice and humans, while supplementing eNAMPT-containing EVs could improve physical activity and extend mouse life span 48. Furthermore, the impact of gut microbiota on human health has recently attracted much attention that may involve membrane vesicles. Shen et al. reported that administration of outer membrane vesicles (OMVs) isolated from *Bacteroides fragilis* could deliver commensal molecules, which mimic the benefits of microbiota 49. This finding was confirmed by other studies in which bacteria-derived OMVs have been shown to effectively modulate host responses, and activate signaling events through the intestinal epithelial barrier 50.

In summary, in response to various stimuli, EVs could be released by various cell types as well as bacteria to modulate the function of near or distant cells. EVs provide an alternative mode of paracrine and endocrine communications compared to the conventional chemical signaling for intercellular communications including direct cell-cell contact or receptor-mediated recognition of soluble hormones and cytokines 43. It is still unclear if EVs can serve as a specialized messaging system in the body. However, specific molecules on the surface membrane of EVs may serve as a special “bar code” recognized by their distantly located special receptors. A recent study reported that cytokines present on the EV surface could target distant recipient cells that express appropriate cytokine receptors 43. Taken together, the direct interaction between EV surface molecules and receptors on the target cells allows EVs to specifically interact with target cells. Although there are still many unanswered questions that need to be addressed, a growing body of research on EVs is expected to generate new information that would provide a better understanding of this field.

## EVs and Adipose Tissues

### Adipose-derived EVs serve as novel adipokines

**Figure 1:**
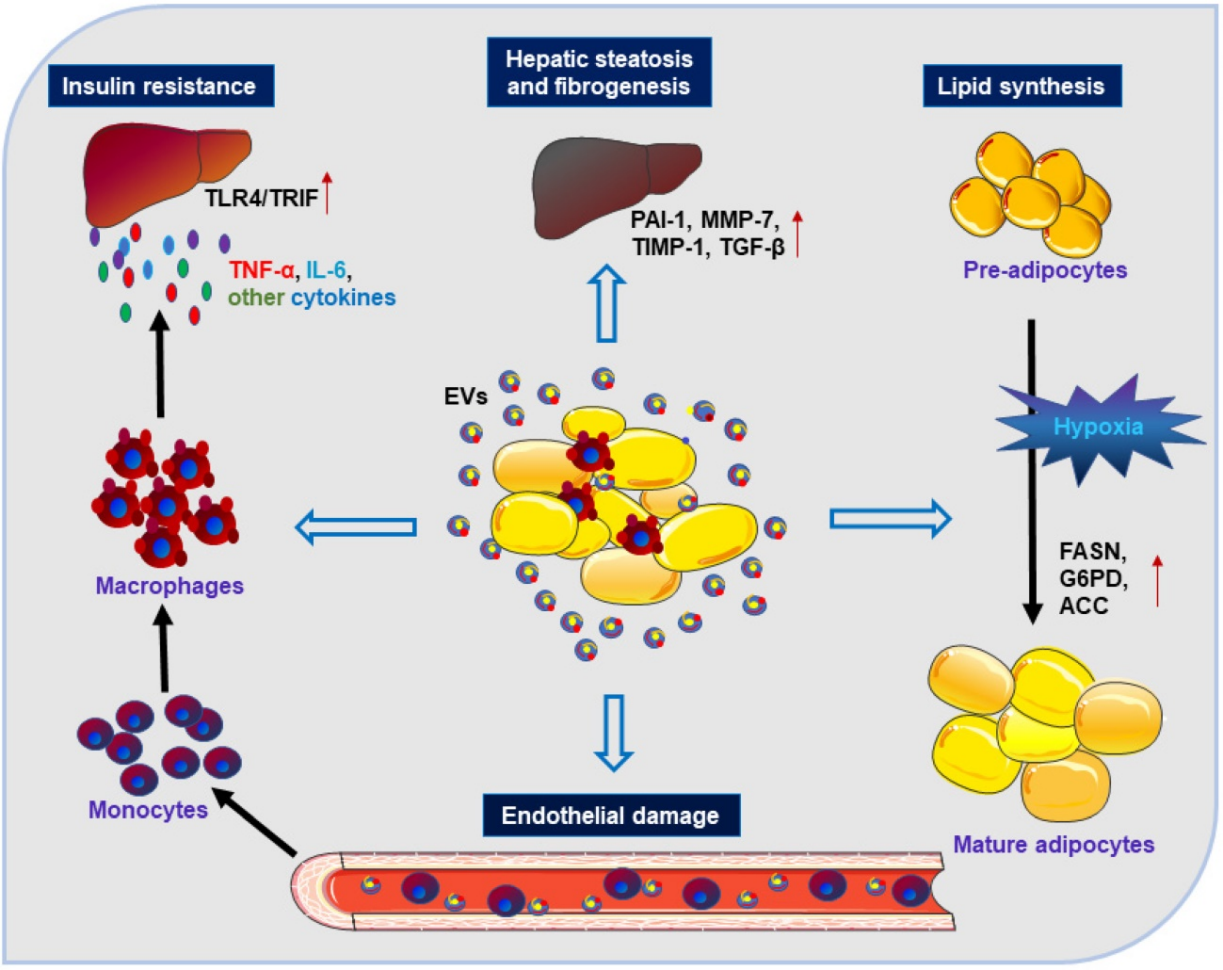
Adipose-derived EVs can function as novel adipokines. Under stimulation by adipocyte-derived EVs, monocytes can transform into activated macrophages with the induction of IR via releasing inflammatory cytokines (such as TNF-α, IL-6, TLR4/TRIF). EVs from WAT can act on the liver and lead to hepatic steatosis and fibrogenesis via involvement of TGF-β, PAI-1, MMP-7, and TIMP-1. EVs derived from adipocytes can impair endothelial function and promote the development of obesity-related metabolic diseases. EVs secreted by hypoxic adipocytes favor the expression of lipogenic enzymes (such as FASN, G6PD, and ACC) that can promote lipid synthesis.

Clinical studies have shown that gastric bypass surgery and the subsequent weight loss can improve insulin resistance (IR) to maintain glucose homeostasis and this beneficial effect might be mediated by circulating adipocyte-derived exosomes 51. AT-derived EVs have been reported to mediate the endocrine link between maternal AT and fetal growth, and to be responsible for fetal overgrowth 52. Besides, AT-derived EVs under hypoxic conditions might promote lipid synthesis by increasing the levels of lipogenic enzymes, including fatty acid synthase (FASN), glucose-6-phosphate dehydrogenase (G6PD) and acetyl-CoA carboxylase (ACC), which may reflect metabolic stress in adipocytes 53. These studies have suggested the potential role of EVs as adipokines contributing to adipose tissue homeostasis or dysfunction (**Figure 1**). Furthermore, AT-derived EVs can also aggravate IR by stimulating monocyte differentiation and macrophage activation, as well by releasing tumor necrosis factor-α (TNF-α) and interleukin-6 (IL-6) 54. Exosomes isolated from visceral adipose tissue (VAT) of obese patients could be integrated into hepatocytes resulting in dysregulation of transforming growth factor-beta (TGF-β) pathway, and promoting the development of NAFLD 55. Besides, AT-derived EVs could also contribute to liver fibrogenesis by extracellular matrix (ECM) accumulation in the liver involving plasminogen activator inhibitor (PAI-1), matrix metalloproteinase (MMP)-7, and tissue inhibitors of metalloproteinase (TIMP)-1 56, 57.

Thus, AT-derived EVs may serve as adipokines to modulate metabolic dysfunction through regulation of adipose tissue homeostasis, promotion of adipose inflammation, or interference with the normal signaling pathways of liver and occurrence of hepatic inflammation and even liver fibrosis. These studies may help us gain a better understanding of the role of AT-derived EVs in the cross-talk between AT and other metabolic organs in the context of obesity.

### Brown adipose tissue (BAT)-derived EVs and white adipose tissue (WAT) browning

Adipose tissues can be classified as white adipose tissue (WAT) and brown adipose tissue (BAT). WAT stores excessive energy in the body, while BAT generates heat under cold stress (non-shivering thermogenesis) with the mediation of uncoupling protein 1 (UCP1), a transmembrane protein in the mitochondrial inner membrane in brown adipocytes 58. The importance of BAT in human metabolism has been demonstrated and its amount was reported to be inversely correlated with body mass index 14. Since mass and activity of BAT decrease with age, it is worthwhile to explore reversing this adverse progression. Remarkable findings from several groups showed that there is a continuum between BAT and WAT in rodents, in which cold exposure or stimulation with adrenergic agonist may be important for elevated UCP1 expression in WAT 59, 60. Interestingly, BAT can secret EVs for communication with other metabolic organs. A recent study by Jung et al. reported that human adipose-derived stem cells (HASCs) could be differentiated into beige/brown adipocytes by EVs derived from HASCs generated during beige adipogenic differentiation 61. Applying these EVs* in vivo* attenuated high-fat diet (HFD)-induced hepatic steatosis and improved glucose intolerance through browning of the adipose tissue in mice 61.

Thomou et al. studied the role of circulating miRNAs in AT by generating mice with adipose tissues deficient in miRNA-processing enzyme Dicer (DicerKO) 62 and found that DicerKO mice exhibited lipodystrophy, BAT whitening, and IR, with reduced circulating miRNAs. Transplantation of wild-type BAT into DicerKO mice, on the other hand, could improve glucose tolerance and reduce fibroblast growth factor-21 (FGF21) in the liver and serum, in which EV-associated miRNAs from BAT might act as novel forms of adipokines to distantly regulate gene expression in the liver 62. These novel findings suggest that, in addition to promoting energy expenditure, BAT-derived EVs also play a role in metabolism, i.e. WAT browning, and cross-talk with the distant organ, the liver 62. Other investigations that analyzed the miRNA expression profile of human subcutaneous adipose tissue during adipocyte differentiation, suggested a close cross-talk between adipogenesis and miRNAs 63, 64.

Overall, the above promising studies indicate that EVs derived from beige/brown adipocytes have beneficial effects on WAT browning, thus providing insights into the development of potential therapeutic strategies for future treatment of obesity and metabolic disorders. Since many of the studies are from rodents, additional investigations in humans are necessary to determine the positive and negative functions of AT-derived EVs. Furthermore, adipogenic miRNAs may serve as biomarkers and therapeutic targets for obesity and its related complications 63, 64.

### Interaction between EVs derived from other cell types and AT

EVs derived from other cell types, such as leukocytes, erythrocytes, platelets, and hepatocytes, may also influence AT and its metabolism 65, 66. A cross-sectional and longitudinal cohort study found that levels of erythrocyte-derived EVs were elevated in individuals with diabetes, and internalization of these diabetic EVs by leukocytes altered their function resulting in secretion of proinflammatory cytokines 67. Furthermore, hepatic EVs were also metabolically active and were involved in oxidative stress, endothelial dysfunction, and drug-induced liver injury 68, 69. Our earlier work demonstrated that tobacco smoke exposure could induce the release of potent collagenolytic MMP-14-containing EVs from cultured macrophages 29. Subsequently, the macrophage-derived collagenolytic EVs were found to contribute to the expansion of AT through degradation of pericellular collagenous web around adipocytes 70. These findings may help explain the abdominal obesity in smokers 70.

**Figure 2:**
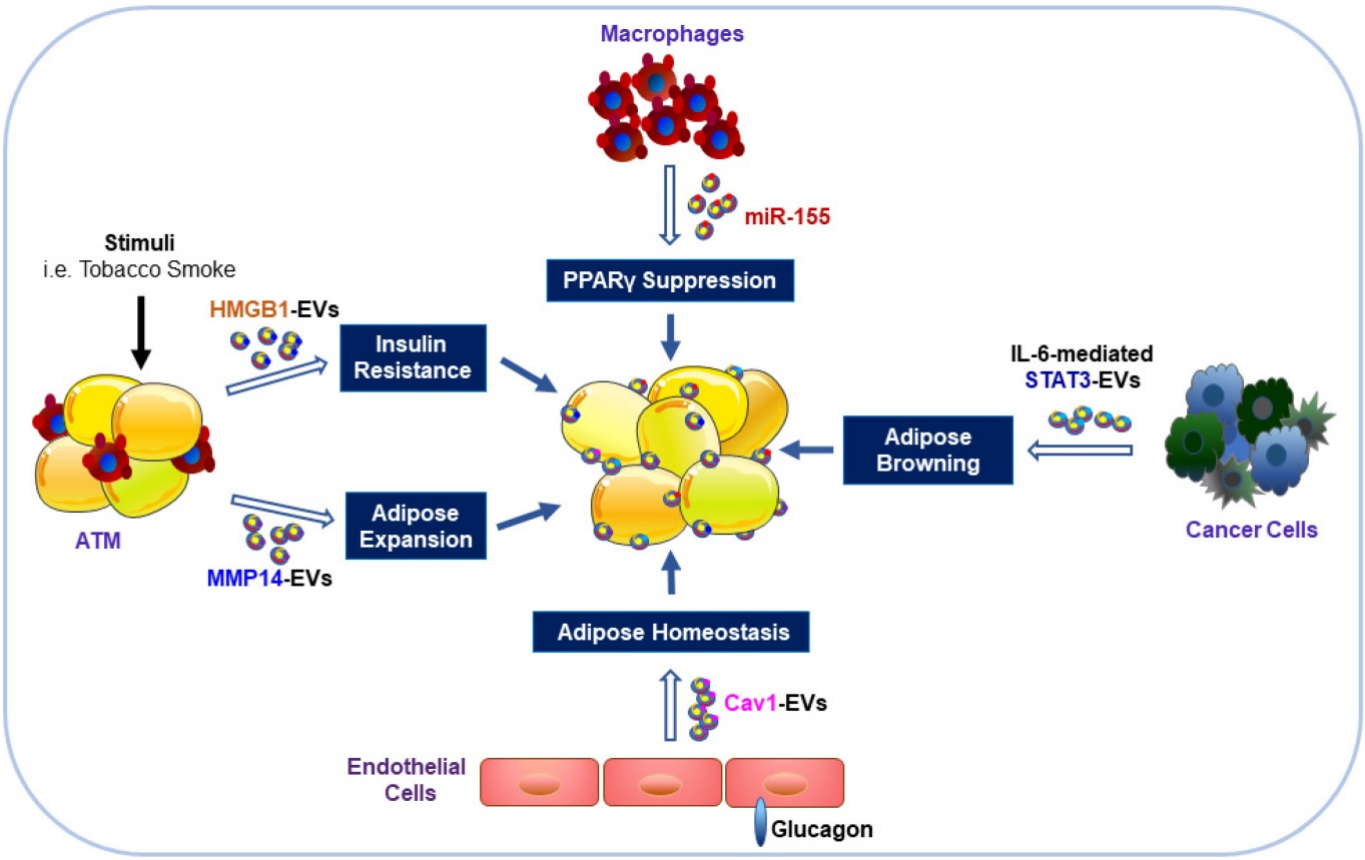
Impacts of EVs derived from other cell types on adipocytes. Exposure of ATM to stimuli (such as tobacco smoke) can result in the release of EVs with HMGB1 and MMP14 that contribute to IR and expansion of the size and volume of adipocytes. EVs from obese ATM harbor miR-155 and can impair insulin signaling and metabolic homeostasis through inhibition of PPARγ gene expression in AT. Endothelial cells stimulated by glucagon can transfer EV-associated cav1 into adipocytes to modulate adipose homeostasis. EVs released from cancer cells can promote WAT browning and lipolysis via activation of the IL-6/STAT3 signaling pathway.

Caveolin 1 (cav1) is an important membrane-bound structural and signaling protein, which is highly abundant in adipocytes and endothelial cells (ECs) 69. Interestingly, Crewe et al. found abundant cav1 protein expression in adipocytes even though it had been specifically ablated 71. By using genetically engineered mouse models, these investigators found that ECs could transfer their released EVs with cav1 to adipocytes that reciprocated by releasing EVs to ECs 71. These cav1-associated EVs were important for not only adipose homeostasis but also systemic metabolic state 71. Taken together, the above studies demonstrated that EVs from non-adipose tissues, i.e. erythrocytes, macrophages, endothelial cells, or hepatocytes are important in whole-body adipose tissue homeostasis (**Figure 2**). Long distance communication between other organs/tissues and the adipose tissue may be mediated through secretion of EVs. However, there are still many questions that need to be addressed and the detailed mechanisms investigated.

Besides the role of EVs in adipose homeostasis, miRNA-containing EVs obtained from ATM of obese mice could induce glucose intolerance and IR when injected into lean mice 71. Conversely, the administration of the ATM EVs isolated from lean mice could reverse the negative changes in obese recipients 71. Our recent work demonstrated that tobacco smoke exposure of macrophages might release EVs with high mobility group box 1 (HMGB1) 72, which could directly impair insulin signaling in cultured adipocytes 12. These results may help explain the adverse effects of tobacco smoking on insulin signaling impairment 73. Furthermore, the overexpressed miR-155 in EVs from obese ATM suppressed gene expression of its downstream peroxisome proliferator-activated receptor γ (PPARγ) in insulin target organs, including AT, liver and muscle, through paracrine or endocrine mechanisms, leading to impaired insulin sensitivity and glucose homeostasis 74. In contrast, EVs from ADSCs could trigger WAT browning and attenuate inflammation by induction of anti-inflammatory M2 macrophage polarization, alleviating obesity and hepatic steatosis with improved insulin sensitivity 75.

In summary, EVs, released from cells other than adipocytes, carry cargos including miRNAs and other bioactive proteins, and are involved in modulation of adipose homeostasis and metabolic states. These studies suggest that investigations on EVs provide insights into the understanding of metabolic systems for the development of novel therapeutic strategies and better treatments of metabolic diseases.

## EVs serve as mediators for intergenerational transmission of metabolic disease risk

Numerous studies have demonstrated that paternal/maternal metabolic disease risk can be transmitted from parents to offspring 76-79. Although genomic DNA transmits inheritance, recent studies demonstrated that epigenetic information also contributes to the non-genetic intergenerational transmission of disease risk to future generations 80, 81. Chen and co-workers reported that injection of sperm tRNA-derived small RNAs (tsRNAs) from male mice with HFD into normal zygotes generated metabolic disorders with altered gene expression of metabolic pathways in early embryos and islets of F1 offspring 82. This novel observation was confirmed by a later study 83, and the changes were independent of DNA methylation at CpG-enriched regions 81, 82. Another study showed that the tsRNAs were transferred to the sperm via epididymal EVs 84. Thus, the evidence suggested that EVs contribute to the intergenerational transmission of the metabolic disease risk from paternal/maternal exposures to their future generations 84-86 and function as mediators of intracellular communication in the mammalian reproductive system 87-90. The studies also indicated that EVs enable external factors from environmental exposure to reach gametes or zygote, as well as the tissues of the maternal reproductive tract, tracking parental exposure for the offspring 87-90.

Chan et al. reported that reproductive tract EVs could transmit information regarding stress in the paternal environment to sperm, potentially altering fetal development 89, and the changes in protein and miRNA content of EVs were long-lasting, suggesting a sustainable programmatic change in response to chronic stress 89. Thus, in sperm maturation, EVs can perform a role in the intergenerational transmission of paternal environmental experience, providing direct communication between somatic cells and germ cells 88, 89, 91. However, these observations raise an interesting question regarding the mechanism of EVs' involvement in the intergenerational transmission of paternal environmental experience? Prevailing data suggest that EVs transfer proteins, lipids, and small RNAs from the epididymal fluid to sperm, promoting sperm motility and oocyte recognition 92. Importantly, the EV cargos, especially small RNAs, exhibit dramatic changes in response to environmental conditions such as smoking, drugs, or dietary constraints, and these environmental signals of paternal experience can be dynamically transferred to sperms by EVs 93-95. Chan et al. reported that alterations of miRNA and protein content of epididymal epithelial cell-derived EVs in response to chronic stress were transferred to sperm transmitting the information from paternal cells to offspring 89. Other small noncoding RNAs could also be transferred to sperm by epididymal EVs. The tsRNAs accounted for 80% of the small RNA content of sperm in the cauda epididymis as per small RNA-sequencing 84. As the levels of the specific tsRNAs were affected in the zygote by low protein diet, the gene expression related to a metabolic phenotype was also altered in the offspring of the protein-restricted males 84.

Collectively, these novel findings support the role of EVs as a vector in transmitting paternal environmental exposure (for eg. HFD 84) and encoding these experiences to sperm for delivery to the offspring 84,89. These studies helped us understand the intergenerational transmission of metabolic disease risks, and may provide insights into the development of new treatments.

## EVs and metabolic diseases

EVs are known to be involved in various human diseases, including obesity and metabolic disorders. Circulating EVs and EV-associated bioactive molecules, including miRNAs, reflect the characteristics of the parental cells and are ubiquitously present in a variety of human biofluids. Therefore, EVs have been proposed to be novel diagnostic and prognostic biomarkers in metabolic diseases 96. One such example is of Perilipin A present in circulating EVs derived from adipocytes that represented a novel biomarker of AT stress 97. Other studies have reported elevated levels of circulating EVs in obesity and its related metabolic disorders, i.e. IR, diabetes, and NAFLD 98-100. Given the clinical relevance and pathological involvement of EVs in IR, diabetes, and NAFLD, we provide a more detailed discussion of EVs in these metabolic diseases in the following.

### EVs and IR

As a major feature of T2DM, IR caused by impaired insulin signaling pathway is relevant to the development of hypertension and atherosclerosis. Freeman and co-workers recently reported higher plasma EV concentrations in patients with DM and its positive association with homeostasis model assessment (HOMA)-IR 67. They found several insulin-signaling proteins in the circulating EVs, indicating that IR may contribute to higher plasma EV levels 67. In contrast, EVs originating from different tissues/organs, including AT and muscle, may be involved in the development of IR 101. EVs isolated from insulin resistant mice could modulate insulin signaling in pancreatic β-cells 102 or skeletal muscle 103, suggesting a role for EVs in insulin signaling in mice.

EVs derived from human subcutaneous or visceral fat tissue may impair insulin signaling in hepatocytes by inhibiting insulin-induced Akt phosphorylation, thus contributing to systemic IR development 104, 105. Yu et al. reported that adipocyte-derived exosomal miR-27a may induce IR in skeletal muscle by suppressing PPARγ expression 104. These studies demonstrated the potential role of adipocyte-derived EVs in IR development by cross-talking between adipose tissue and insulin sensitive organs, like liver and skeletal muscle 102, 104-107. Interestingly, EVs released from hypoxic adipocytes and obese individuals can impair insulin-stimulated uptake of 2-deoxyglucose in adipocytes, suggesting that EVs act as mediators for transferring hypoxia-induced IR signatures within the adipose tissue 106. Furthermore, adipocyte-derived EVs also lead to IR by down-regulating expression of insulin receptor substrate-1 (IRS-1) and hormone-sensitive lipase (HSL) in adipocytes 108. In addition to the role of adipocyte-derived EVs, muscle EVs from mice with HFD-induced IR can integrate into the pancreas *in vivo* and modulate gene expressions in cultured β-cells and isolated islets *in vitro*, causing β-cell proliferation, thus explaining adaptations in beta cell mass during IR 102. Consistent with this study, skeletal muscle has recently been shown to be an active endocrine organ that secretes myokines and contributes to the development of IR 107.

Obesity is characterized by chronic low-grade inflammation, which leads to the development of IR 109. Adipocytes and AT-derived EVs may differentiate monocytes to a phenotype of AT macrophages and contribute to AT inflammation and IR 54. Indeed, EVs from AT of HFD-induced mice when injected into control mice could increase the levels of inflammatory factors, including IL-6 and TNF-α, leading to the development of IR, via TLR4 pathway as this effect was attenuated in TLR4 KO mice 54. A recent study demonstrated that EVs isolated from cultured 3T3-L1 adipocytes could induce pro-inflammatory M1 polarization of macrophages through activation of Ptch/PI3K signaling pathways by the EV-associated sonic hedgehog protein 110. These results demonstrated that adipocyte-derived EVs can modulate IR development by affecting macrophage inflammatory responses. Conversely, macrophage-derived EVs can also contribute to the IR development through different mechanisms. Exosomal miR-155 secreted by AT macrophages of obese mice has been shown to impair Akt phosphorylation and repress PPARγ expression, thus aggravating IR in insulin target organs such as AT, skeletal muscle, and liver 74. Our recent study demonstrated that macrophage-derived EVs carry HMGB1, a nuclear nonhistone DNA-binding protein that functions as a proinflammatory cytokine and can directly impair insulin signaling in cultured adipocytes *in vitro* 12. We had previously reviewed the association between EVs and insulin sensitivity 19.

Hepatokines are liver-derived proteins, including fetuin A, fetuin B, retinol-binding protein 4 (RBP4) and selenoprotein P, which are associated with the induction of metabolic dysfunction 39, 61. Contrary to the harmful effects of cytokines on IR and glucose dysregulation in obesity and NAFLD, recent studies indicated that type I interferon (IFN) may protect against metabolic dysfunction 108, 111; other studies reported that IFNs are associated with EVs in various pathophysiological conditions 43, 112, 113. Our laboratory recently provided evidence that EV-associated mitochondrial antiviral signaling (MAVS) triggered IFNβ production from dendritic cells 112. However, it has not been evaluated if EV-associated IFNα or IFNβ can protect against metabolic dysfunction.

Overall, published literature underscored that EVs derived from adipocytes, skeletal muscles, or adipose tissue infiltrated macrophages and might contribute to IR development by interfering insulin signaling in insulin sensitive organ/tissues or causing pancreatic β-cell dysfunction. These results identify EVs as potential future therapeutic targets for the management of IR and metabolic syndromes.

### EVs and type 1 diabetes mellitus

The appearance of diabetes can be attributed to the dysregulated crosstalk between endocrine organs, such as AT, liver, and skeletal muscles, that are involved in the development of metabolic diseases 54. So far, we have discussed that EVs may play an important role in the pathogenesis of IR; here, we expand the discussion to the effects of EVs on the development of T2DM. Recent studies also suggested that EVs may contribute to the etiology of type 1 diabetes mellitus (T1DM) 113, 114.

T1DM is a disease characterized by defective insulin synthesis as a consequence of auto-immunologic injury of pancreatic β-cells, which accounts for 5-10% of patients with diabetes 115. A growing body of literature has shown multiple roles of EV-associated miRNAs in T1DM 114, 116, 117. A recent study by Guay and co-workers found that rodent and human T lymphocyte-derived EVs containing miR-142-3p, miR-142-5p, and miR-155 could be transferred to β cells resulting in apoptosis and contributing to the development of T1DM 117. Furthermore, Rutman et al. demonstrated that EVs from human islets of Langerhan cells could activate B cells in T1DM patients to produce antibodies against glutamic acid decarboxylase 65 (GAD65), an early marker for beta cell destruction 116. Interestingly, a recent study also demonstrated that the initial autoimmune response in T1DM was induced by exosomal β-cell autoantigens, such as GAD65 and IA-2 in response to endoplasmic reticulum stress 118. Therefore, lymphocyte-derived EVs can induce β cell damage, while destructive islet cells can release GAD65-containing EVs that trigger B cells to produce GAD65 auto-antibody, indicating the role of EVs in the etiology and development of T1DM. Besides their pathogenic effects, EVs may also serve as biomarkers in T1DM. Lakhter et al. observed that cultured β-cells could release miR-21-5p with EVs under cytokine stimulation *in vitro*, and serum EV-derived miR-21-5p was increased threefold in children with new-onset T1DM compared with healthy children 119.The authors proposed that circulating EV-associated miR-21-5p may serve as a biomarker for the development of T1DM 119. Another study assessed miRNAs expression in urinary EVs from individuals with T1DM and found enrichment of miR-130a and miR-145, while miR-155 and miR-424 were reduced 120. These findings provide compelling evidence that EVs bearing miRNAs can be involved in the pathological process of T1DM, probably through transferring to β cells to cause apoptosis or induce autoimmune response. In addition, compared with healthy individuals, miRNAs in serum and urinary EVs from T1DM patients show different levels, which indicate EVs containing miRNAs may act as a novel marker of T1DM in the future.

### EVs and type 2 diabetes mellitus

T2DM is characterized by relative insulin deficiency due to a progressive insufficiency in insulin secretion in individuals with IR, which accounts for 90-95% of patients with diabetes 104. A meta-analysis of 48 studies demonstrated elevated circulating EVs derived from endothelium, platelets, and monocytes in T2DM patients as compared to those in controls 121, suggesting a potential role of EVs in the pathogenesis of T2DM or its complications. Fu and co-workers reported that hepatocellular EVs derived from HFD-induced obese mice promoted islet β cell compensatory hyperplasia in obesity and insulin resistance 122. Jalabert et al. indicated that EVs released from HFD-induced, insulin-resistant muscles could cause downregulation of Ptch1, a negative regulator of Hedgehog signaling in pancreatic development 102. Thus, either hepatocyte- or muscle-derived EVs may act distantly to influence the β-cell mass during the development of IR and T2DM 102, 122. Also, various studies, including ours 123, have demonstrated that EV-associated molecules may impair insulin signaling in cultured adipocytes 11, 19, 72, 110, 123. Isolated circulating exosomes, but not MVs, from patients with metabolic syndromes could also decrease insulin signaling in cultured hepatocytes 124.

Furthermore, several studies have demonstrated that EVs are associated with the pathogenesis of T2DM and contribute to the development of its related complications 125, 126. Rossi et al. reported that water channel aquaporins (AQPs) 5 and 2 expressed on the plasma membrane of epithelial tubular cells were significantly increased in diabetic nephropathy (DN) patients as well as in the urine EVs from 35 diabetic patients 127. Interestingly, urinary EV-associated AQP5 was also correlated with the histological grade of diabetic nephropathy (DN), thus may serve as novel noninvasive biomarkers in classifying the clinical stage of DN 127. Interestingly, IR has been shown to cause diminished glucose uptake in similar regions of the brain in patients with Alzheimer's disease and T2DM. In this context, Kapogiannis et al. have shown that neural-derived blood EVs carry insulin receptor substrate 1 (IRS-1) in patients with preclinical Alzheimer's disease 128.

Collectively, EVs appear to exert crucial roles in T2DM development via interfering with pancreatic islet mass homeostasis, or modulating insulin signaling in adipose tissues or liver. EVs may also contribute to the complications of T2DM. Thus, EVs might be novel therapeutic targets for the treatment of IR and protection of β-cell dysfunction during the development of T2DM 101,114.

### EVs and NAFLD

NAFLD encompasses a broad spectrum of conditions, including isolated hepatic steatosis, NASH, and cirrhosis 129. With the accumulation of certain toxic lipids in cells, lipotoxicity-associated hepatocyte damage is considered as one of the key events that promote NAFLD progression 130. Interestingly, toxic lipids including palmitate (PA) and lysophosphatidylcholine (LPC) could stimulate EVs release from hepatocytes of rodent animals and humans 122,131,132. Recent publications suggest that EVs play an important role in the physiology and pathophysiology of liver diseases 133. EVs derived from lipotoxic hepatocytes might promote hepatic inflammation, angiogenesis, and fibrosis as multiple-hit mechanisms of NAFLD pathogenesis 134. EV release by LPC was mediated by Rho-associated, coiled-coil-containing protein kinase 1 (ROCK1) and tumor necrosis factor-like apoptosis-inducing ligand (TRAIL) receptor 2 (TRAIL-R2) signaling cascade, including TRAIL-R2, caspase 8 and caspase 3 132. Administration of ROCK1 inhibitor could reduce the release of hepatocyte-derived EVs containing TRAIL, leading to attenuated liver injury, inflammation, and fibrosis 132.

In NAFLD, the damaged hepatocytes induced the infiltration and activation of macrophages and other immune cells important for the development of inflammation 132, 134. Ibrahim et al. found C-X-C motif chemokine 10 (CXCL10) as another protein cargo on LPC-induced EVs and proposed that MLK3 could induce lipotoxic hepatocytes to release CXCL10-enriched EVs, which were chemo-attractive toward macrophages in vitro, while MLK3^-/-^ mice were protected against the development of dietary steatohepatitis 135, 136. Hepatocytes cultured with PA could also release EVs enriched in C16:0 ceramide in an inositol-requiring enzyme 1α (IRE1α)-dependent manner and the macrophage chemotaxis could be activated by the ceramide metabolite, sphingosine-1-phosphate (S1P) 137. Furthermore, Bruno et al. demonstrated that lipotoxic injury-induced EVs from hepatocytes could stimulate activation of M1 macrophages through EV-associated miR-192-5p, the blood levels of which positively correlated with hepatic inflammatory activity score and disease progression in NAFLD patients 138. Interestingly, mice and patients with NASH showed high plasma levels of EV-associated mitochondrial DNA, which could selectively upregulate TNF-α through activation of TLR9, while removal of these EVs or treatment with TLR9 antagonist blocked the development of NASH 139. Therefore, it is plausible that hepatocyte-derived EVs can contribute to the NAFLD through macrophage recruitment and activation, or induction of proinflammatory cytokines 135-139. NAFLD progression is characterized by liver inflammation and fibrosis after repeated and sustained injuries, leading to end-stage liver diseases, such as cirrhosis and hepatocellular carcinoma.

**Figure 3:**
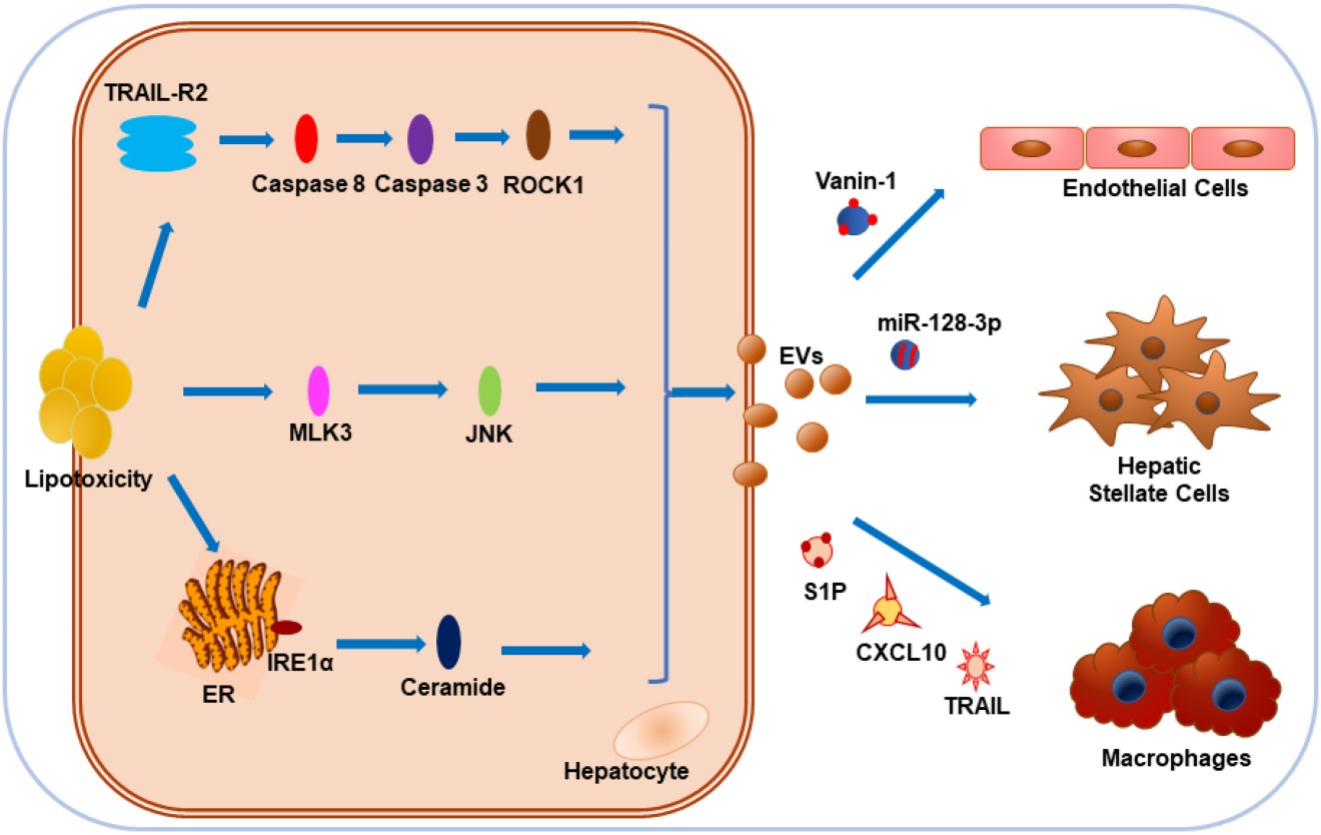
Impact of hepatocyte-derived EVs on NAFLD under lipotoxicity. Hepatocytes tend to release EVs in response to toxic lipids, including PA and LPC. Lipotoxicity-induced EV release is dependent on TRAIL-R2 signaling, stress kinase MLK3, and ER stress sensor IRE1α. CXCL10 and ceramide-bearing EVs mediate monocyte/macrophage chemotaxis to hepatocytes while TRAIL-laden EVs activate macrophages. Vanin-1-enriched EVs can mediate endothelial cell migration, while miR-128-3p-bearing EVs contribute to the proliferation and activation of HSCs.

Povero et al. found that hepatocyte-EV-associated miR-128-3p might be efficiently internalized by hepatic stellate cells (HSCs) in response to lipotoxicity, thus facilitating the development of liver fibrosis by repressing PPARγ expression 140. Also, with the stimulation of lipid-induced toxicity, hepatocyte-derived EVs display pro-angiogenic features and are internalized by endothelial cells via vanin-1, a surface cargo protein 131. To sum up, it appears that during lipotoxicity, various contents of EVs originating from hepatocytes, such as proteins, miRNAs, and mitochondrial DNA, exert a crucial impact on NAFLD progression by influencing hepatic macrophages, liver fibrosis, or angiogenesis. More research in a clinical setting is required to test this hypothesis (**Figure 3**).

## Conclusions and Future Perspectives

Herein, we have summarized the recent literature depicting the role of adipocyte-derived EVs in several metabolic diseases. Numerous studies reported that WAT may contribute to metabolic disorders, while the browning WAT and BAT exerted beneficial effects. In recent years, EVs have attracted much attention for their role in metabolic dysfunction, in particular obesity and its complications. Studies have demonstrated that EVs derived from many cell types serve as novel mediators for long-distance communication between various cells and organs, and are involved in the development of obesity-associated metabolic disorders, including IR, diabetes, and NAFLD. Interestingly, EVs have been shown to transfer transgenerational information of metabolic disease risks from parents to the offspring in an epigenetic fashion, challenging the classical concept of genetic transmission across generations. Acting as novel mediators and biomarkers in the crosstalk between organs, EVs are crucial for maintaining metabolic homeostasis and regulating metabolic disorders. Investigation of the pathophysiology of EVs provides new opportunities in diagnosing and combatting metabolic disorders. However, there remain many outstanding challenges in the field. For example, the lack of specific, unambiguous EV markers and the dearth of adequate EV isolation methods represent serious limitations in the EV research field. Advanced investigations in analyzing the physical, chemical, and biological features of EVs may help further our understanding of EV functions and applying the relevant knowledge in clinical practice. Furthermore, the investigations of the role of EVs in the pathophysiologic processes of human diseases and the underlying molecular mechanisms are still preliminary. Last but not least, clinical research as well as applications of EVs in clinical practice is still limited. Therefore, further research in this field is warranted. Ongoing investigations are expected to provide insights into the role of EVs in metabolic homeostasis and disorders, and, in the future, afford powerful tools for the applications of EVs in diagnosis, treatment, and prognosis of metabolic diseases.

## Abbreviations

ACC: acetyl-CoA carboxylase
AdicerKO: knockout of the miRNA-processing enzyme Dicer
ADSCs: Adipose-derived stem cells
AQPs: Aquaporins
ATM: Adipose tissue macrophages
BAT: Brown adipose tissue
CAC: Cancer-related cachexia
Cav1: Caveolin 1
CCN2: Connective tissue growth factor
CD: Chow diet
CTLA4: Cytotoxic T-lymphocyte antigen 4
CXCL10: C-X-C motif chemokine 10
DAMPs: Danger-associated molecular patterns
DN: Diabetic nephropathy
ECs: Endothelial cells
ECM: Extracellular matrix
Enampt: Extracellular nicotinamide phosphoribosyltransferase
ESCRT: Endosomal Sorting Complex Required for Transport
EVs: Extracellular vesicles
FASN: fatty acid synthase
GAD65: glutamic acid decarboxylase 65
GC: gastric cancer
G6PD: glucose-6-phosphate dehydrogenase
GSDMD: gasdermin D
HFD: high-fat diet
HIF: hypoxia-inducible factor
HMGB1: high mobility group box 1
HOMA: homeostasis model assessment
HPA: hypothalamic-pituitary-adrenal
HSCs: hepatic stellate cells
HSL: hormone sensitive lipase
IL-18: interleukin-18
IL-1β: interleukin-1β
IL-6: interleukin-6
IR: insulin resistance
IRS-1: Insulin receptor substrate 1
LLC: lung carcinoma
LPC: lysophosphatidylcholine
MAVS: mitochondrial antiviral signaling
MLK3: mixed lineage kinase 3
MLKL: mixed lineage kinase domain-like
MMP: matrix metalloproteinasse
MVs: microvesicles
NAFLD: non-alcoholic fatty liver disease
NASH: non-alcoholic steatohepatitis
PA: Palmitate
PAI-1: plasminogen activator inhibitor
PAR: protease-activated receptor
PBMC: peripheral blood mononuclear cell
PC: pancreatic cancer
PDAC: pancreatic ductal adenocarcinoma
PMFs: portal myofibroblasts
PPARγ: peroxisome proliferator-activated receptor γ
PS: phosphatidylserine
RIPK3: receptor-interacting protein kinase-3
ROCK1: rho-associated, coiled-coil-containing protein kinase 1
SAT: subcutaneous adipose tissue
S1P: sphingosine-1-phosphate
STAT3: transcription 3
T1DM: type 1 diabetes mellitus
T2DM: type 2 diabetes mellitus
TF: tissue factor
TIMP: tissue inhibitors of metalloproteinase
TLR4: Toll-like receptor-4
TNF-α: tumor necrosis factor-α
TRIF: Toll-interleukin-1 receptor domain-containing adaptor protein inducing interferon-β
tsRNAs: tRNA-derived small RNAs
TSE: tobacco smoke extracts
TGF-β: transforming growth factor beta
TRAIL: tumor necrosis factor-like apoptosis inducing ligand
TRAIL-R2: tumor necrosis factor-like apoptosis inducing ligand receptor 2
UCP1: uncoupling protein 1
VAT: visceral adipose tissue
VEGF-A: vascular endothelial growth factor A
WAT: white adipose tissue
